# Parameters of Right Ventricle as etiological distinction between ischemic and non-ischemic dilated cardiomyopathy

**DOI:** 10.1186/1532-429X-17-S1-P311

**Published:** 2015-02-03

**Authors:** Hélder Andrade Gomes, Mariana M Lamacié, Fábio V Fernandes, Bernardo N Abreu, Matheus D Freitas, Paulo C Dias Filho, Valéria Moreira, Adriano Carneiro, Tiago A Magalhães, Carlos E Rochitte

**Affiliations:** 1 Hospital do Coração, São Paulo, Brazil

## Background

Dilated left ventricular volumes (VE) is one of the main factors of poor cardiovascular prognosis with systolic dysfunction. Myocardial fibrosis detected by late gadolinium enhancement (LGE) on cardiac magnetic resonance (CMR) has been recognized in recent years as an independent prognostic factor in dilated cardiomyopathy, in addition to guiding the etiological diagnosis, and most often the only noninvasive way to differentiate ischemic and nonischemic. The aim of our study was to determine the prevalence of myocardial fibrosis in patients with dilated LV and the morphological and functional differences between ischemic and non-ischemic patterns.

## Methods

We analyzed 114 consecutive patients ≥ 35 years old who were undergoing CMR with LV volume ≥ 95 ml/m2 between March/2013 and August/2014. After identifying the prevalence of myocardial fibrosis, we analysed the morphological and functional differences between patients with ischemic pattern of LGE and non-ischemic pattern of LGE.

## Results

Patients were 57 ± 11 years old, BMI = 27.6 ± 5.0 kg / m2, LVEF = 43 ± 17%, RVEF = 57 ± 13% and 81% male. Sixty-two (54%) had myocardial fibrosis by LGE, including 36 (58%) with ischemic pattern and 25 (40%) with non-ischemic pattern, and one patient was excluded for presenting the two types of LGE. Among patients with myocardial fibrosis, those who exhibited a pattern of non-ischemic LGE had worse RVEF (50 ± 16% vs 62 ± 9%, p = 0.003) and greater RV volumes (RVEDVI/RVESVI 72±21/36±18 vs 59±15/22±8 ml/m2, p = 0.012/0.001), with no significant differences in age, sex, BMI, parameters of LV (volumes and EF) or heart rate during CMR performance. Regarding the number of LV segments affected by fibrosis, ischemic patients had more extensive disease (7.1±4.2 vs 3.7±3.2 segments, p = 0.001).

## Conclusions

Patients with non-ischemic myocardial fibrosis pattern had higher RV volumes and worse RVEF, and myocardial fibrosis in fewer number of LV segments compared to ischemic pattern. Volumes and EF preserved RV might suggest ischemic etiology in patients under investigation for dilated cardiomyopathy.

## Funding

None.

**Table 1:** 

	Ischemic	non-ischemic	p
Patients	36 (59%)	25 (41%)	NA

Age, yo	62 ± 10	59 ± 10	0.229

Gender, male (%)	32 (89%)	19 (76%)	0.214

BMI	27.7 ± 4.2	28.1 ± 4.7	0.765

HR	68 ± 14	69 ± 12	0.732

LVEF	36 ± 14	39 ± 18	0.493

LVEDVI	130 ± 44	125 ± 34	0.645

LVESVI	86 ± 46	81 ± 44	0.637

RVEF	62 ± 9	50 ± 16	0.003*

RVEDVI	59 ± 15	72 ± 21	0.012*

RVESVI	22 ± 8	36 ± 18	0.001*

Segments with LGE	7.1 ± 4.2	3.7 ± 3.2	0.001*

*p < 0.05

